# Relationship between human papillomavirus and serum vitamin D levels: a systematic review

**DOI:** 10.1186/s12879-024-09006-8

**Published:** 2024-01-13

**Authors:** Seyedeh Maryam Khalili, Elnaz Haji Rafiei, Marjan Havaei, Leila Alizadeh, Fereshte Ghahremani, Zohreh Keshavarz, Ali Montazeri, Hedyeh Riazi

**Affiliations:** 1 Students Research Committee, School of Nursing and Midwifery, Shahid Beheshti University of Medical Sciences, Tehran, Iran; 2 Department of Midwifery and Reproductive Health, Midwifery and Reproductive Health Research Center, School of Nursing and Midwifery, Shahid Beheshti University of Medical Sciences, Tehran, Iran; 3 Population Health Research Group, Health Metrics Research Center, Iranian Institute for Health Sciences Research, ACECR, Tehran, Iran; 4 Faculty of Humanity Sciences, University of Science and Culture, Tehran, Iran

**Keywords:** Human papillomavirus, HPV, Vitamin D, Systematic review, Sexual health

## Abstract

**Background:**

Human papillomavirus (HPV) is one of the most prevalent sexually transmitted diseases worldwide. The present review was conducted to accumulate evidence on the relationship between cervicovaginal human papillomavirus infection and serum vitamin D status.

**Methods:**

Electronic databases including Web of Science, Embase, Scopus, and PubMed were searched by different combinations of keywords related to “human papillomavirus” and “vitamin D”, obtained from Mesh and Emtree with AND, and OR operators without any time restriction until December 24, 2022. Selection of articles was based on the inclusion and exclusion criteria. Newcastle-Ottawa Scale was used for quality assessment. The Preferred Reporting Items for Systematic Reviews and Meta-Analyses (PRISMA) checklist was applied for reporting.

**Results:**

In total, 276 citations were retrieved. After removing duplicates, and non-related articles, the full texts of 7 articles were reviewed including 11168 participants. Three studies reported that there was a positive relationship between vitamin D deficiency and cervicovaginal human papillomavirus while three studies did not. One study showed a significant positive association between higher vitamin D stores and short-term high-risk human papillomavirus persistence.

**Conclusions:**

The findings showed no firm evidence for any association between serum vitamin D level and cervicovaginal human papillomavirus infection, although the possible association could not be discarded. Further investigations are needed to reach sound evidence.

## Background

Human papillomavirus (HPV) is one of the most prevalent sexually transmitted diseases (STDs) worldwide [1]. While most HPV infections are cleared spontaneously with the natural immune response, part of this infection persists, which can lead to malignant disease [2]. Persistent oncogenic HPV infections are responsible for cervical and anal cancers due to structural changes in DNA [3, 4]. Cervical cancer is the fourth most common female cancer in women aged 15 to 44 years worldwide [5]. Globally, more than 95% of cervical cancers and more than 300,000 deaths per year occur due to HPV infection [6, 7]. About 341,831 new cervical cancer cases are diagnosed annually in the world and the global mortality rate among women is reported 13.3/100,000 in 2020 [8]. Decreasing the incidence of HPV infections and associated carcinogenicity is possible by understanding the factors contributing to the occurrence and persistence of the infection [9].

In previous studies, the relationship between HPV and some vitamins including vitamin D has been assessed [10, 11]. It is emphasized that vitamin D, in addition to its essential role in maintaining calcium and phosphorus homeostasis and bone health [12], contains antiviral and immunomodulatory effects, and plays a key role in the modulation of the immunological response in infectious diseases [12, 13]. The maturation of macrophages, function regulation, and proliferation of lymphocytes are attributed to this vitamin [14]. Indeed, sufficient vitamin D levels protect against some infectious diseases, while non-optimal levels of this vitamin are associated with an increased risk of incidence and severity of the disease [15, 16]. The effectiveness of vitamin D in the successful treatment of viral hepatitis, respiratory infections, and Herpes virus, is confirmed [17]. These findings are related to the role of vitamin D in strengthening innate and acquired immunity against infection [18].

Although there is progressive evidence suggesting that vitamin D has an important role in the immune system- boosting [13, 14, 16, 19], and immune response is thought to have a critical role in preventing and treating HPV infection, no conclusive evidence is available regarding serum levels of vitamin D in patients with HPV infection. Some previous studies reported lower levels of vitamin D in patients with HPV [20–22], while some other studies observed inconsistent findings [23, 24]. Considering these incompatible reports, the present review aimed to investigate the relationship between cervicovaginal HPV infection and vitamin D serum levels to compile evidence on the topic. Understanding this relationship may ultimately contribute to more effective strategies for preventing HPV-related diseases and improving overall public health.

## Methods

### Design

This was a systematic review of the literature. The Preferred Reporting Items for Systematic reviews and Meta-Analyses (PRISMA) guideline was followed [25].

### Search strategy

Electronic databases including Web of Science, Embase, Scopus, and PubMed were searched. In order to maximize the search comprehensiveness, general keywords including human papillomavirus, vitamin D, 25-hydroxyvitamin D, cholecalciferol, as well as standardized keywords of Emtree and Mesh including papillomavirus infections, Alphapapillomavirus, Human papillomavirus, vitamin D cholecalciferol, and their combination were used through relevant operators (such as AND, and OR). Furthermore, the references of the assessed papers were reviewed manually. The final keywords were reviewed by the research supervisor. An example of the PubMed search query is shown in Table 1.

**Table 1:** An example of PubMed search query

("Alphapapillomavirus" [mh] OR "Human papillomavirus 16" [mh] OR "Human papillomavirus 31" [mh] OR "Human papillomavirus 18" [mh] OR "Human papillomavirus 6" [mh] OR "Human papillomavirus 11" [mh] OR "Papillomavirus Infections" [mh] OR "Warts" [mh] OR Alphapapillomaviruses [tiab] OR HPV Human Papillomavirus [tiab] OR HPV Human Papillomaviruses [tiab] OR Human Papillomavirus [tiab] OR Human Papillomaviruses [tiab] OR HPV 16 [tiab] OR Human papillomavirus type 16 [tiab] OR HPV 31 [tiab] OR Human papillomavirus type 31 [tiab] OR HPV 18 [tiab] OR Human papillomavirus type 18 [tiab] OR HPV 6[tiab] OR Human papillomavirus type 6 [tiab] OR Papillomavirus [tiab] OR HPV 11 [tiab] OR Human papillomavirus type 11 [tiab] OR Papillomavirus Infection [tiab] OR Human Papillomavirus Infection [tiab] OR Human Papillomavirus Infections [tiab] OR HPV Infection [tiab] OR HPV Infections [tiab] OR Wart [tiab]) AND ("Vitamin D" [mh] OR "25-Hydroxyvitamin D 2" [mh] OR "Calcifediol 25-hydroxyvitamin D" [mh] OR "Cholecalciferol" [mh] OR "Hydroxycholecalciferols" [mh] OR "Calcitriol" [mh] OR 25 Hydroxyvitamin D 2 [tiab] OR 25 Hydroxyergocalciferol [tiab] OR 25 Hydroxyvitamin D2 [tiab] OR Ercalcidiol [tiab] OR 25 Hydroxycalciferol [tiab] OR 25 Hydroxyvitamin D 3 [tiab] OR 25 Hydroxycholecalciferol Monohydrate [tiab] OR 25 Hydroxyvitamin D3 [tiab] OR Calcidiol [tiab] OR 25 Hydroxycholecalciferol [tiab] OR Calcifediol Anhydrous [tiab] OR Dedrogyl [tiab] OR Hidroferol [tiab] OR Calderol [tiab] OR 25-hydroxyergocalciferol [tiab] OR Calciol [tiab] OR Vitamin D 3 [tiab] OR Vitamin D3 [tiab] OR Cholecalciferols [tiab] OR Hydroxyvitamins D [tiab] OR Hydroxycholecalciferol [tiab] OR Bocatriol [tiab] OR Calcijex [tiab] OR Calcitriol KyraMed [tiab] OR Calcitriol Nefro [tiab] OR Decostriol [tiab])	

### Selection criteria

Observational studies (cohort, case‑control, and cross‑sectional) published in English that assessed the relationship between HPV infection and vitamin D status in women included until December 24, 2022. The articles were selected based on the inclusion and exclusion criteria, with no restrictions regarding the start date for article inclusion. Review studies, letters to editors, conference papers, non-English articles, and those with unavailable full texts excluded.

### Study selection process

The title, abstract, and full text of articles were reviewed by two authors independently. In the case of meeting the inclusion criteria, the article was included for the quality assessment and data extraction, and in case of disagreement, the third author was consulted.

### Data extraction

Data extraction was done based on a checklist, which included the first author’s name and year of publication, country, study design, sample size, and participants, as well as the most important findings.

### Quality assessment

Two reviewers independently assessed the quality of the selected articles based on the Newcastle-Ottawa Scale (Newcastle-Ottawa cohort scale version and its modified version for cross-sectional studies) [26]. This Scale 3 domains (selection, comparability, and outcome. For case control studies instead of outcome the domain is exposure). The maximum quality score for each type of studies is 9. Studies with a score of 7-9, are considered as high quality, 4-6 as high risk, and 0-3 as very high risk of bias. For cohort studies, a score of 6 or higher is considered as low risk and good quality, and a score of <6 is considered as high risk and low quality [27]. Disagreements between the two reviewers were resolved by discussion with the research supervisor. The result of quality assessment of the selected articles is shown in Table 2.

**Table 2:** Quality assessment of included studies using the Newcastle-Ottawa Scale*

**Assessment of the quality of cohort studies**									
**Selection (max 4 scores)**					**Comparability (max 2 scores)**	**Outcome (max 3 scores)**			Total score**
**Author/ Year/ Reference**	**Representativeness of the exposed cohort**	**Selection of the non-exposed cohort**	**Ascertainment of exposure**	**Demonstration that outcome of interest was not present at start of study**	**Comparability of cohorts on the basis of the design or analysis**	**Assessment of outcome**	**Was follow-up long enough for outcomes to occur**	**Adequacy of follow up of cohorts**	
Chu et al. (2021) [28]	*	*	*	*	**	*	*	*	9
El-Zein et al. (2021) [24]	*	*	*	*	**	*	*	*	9
Troja et al. (2021) [29]	*	*	*	*	**	*	*	*	9
**Assessment of the quality of cross-sectional studies**									
**Selection (max 5 scores)**					**Comparability (max 1 score)**	**Outcome (max 3 scores)**			
**Author/ Year/ Reference**	**Representativeness of the sample**	**Sample size**	**Non-response rate**	**Ascertainment of the measure**	**Potential confounders were investigated based on the study design or subgroup analysis**	**Assessment of the outcome**	**Statistical test**	**-**	**Total score**
Shim et al. (2016) [20]	-	*	-	**	*	**	*	-	7
Garcia-Carrasco et al. (2015) [23]	-	*	-	**	*	**	*	-	7
Troja et al. (2020) [30]	-	*	*	*	*	*	*	-	6
Mertoğlu et al. (2017) [31]	-	-	-	*	-	*	-	-	2
Çakir et al. (2022) [32]	-	-	-	*	-	*	-	-	2
**Assessment of the quality of case-control studies**									
**Selection (max 4 scores)**					**Comparability (max 2 scores)**	**Exposure (max 3 scores)**			
**Author/ Year/ Reference**	**Adequate case definition**	**Representativeness of the cases**	**Selection of controls**	**Definition of controls**	**Basis of the design or analysis**	**Ascertainment of exposure**	**Same method of ascertainment for cases and controls**	**Non-Response rate**	**Total score**
Ozgu et al. (2016) [21]	*	*	*	*	**	-	*	*	8

^*^ The last two studies in cross-sectional section are not included in the review

^**^ Each asterisk is equivalent to one score. The maximum score is 9. Studies with a score of 7-9, are considered as high quality, 4-6 as high risk, and 0-3 as very high risk of bias. For cohort studies, a score of 6 or higher is considered as low risk and good quality, and a score of <6 is considered as high risk and low quality

## Results

**Fig. 1:**
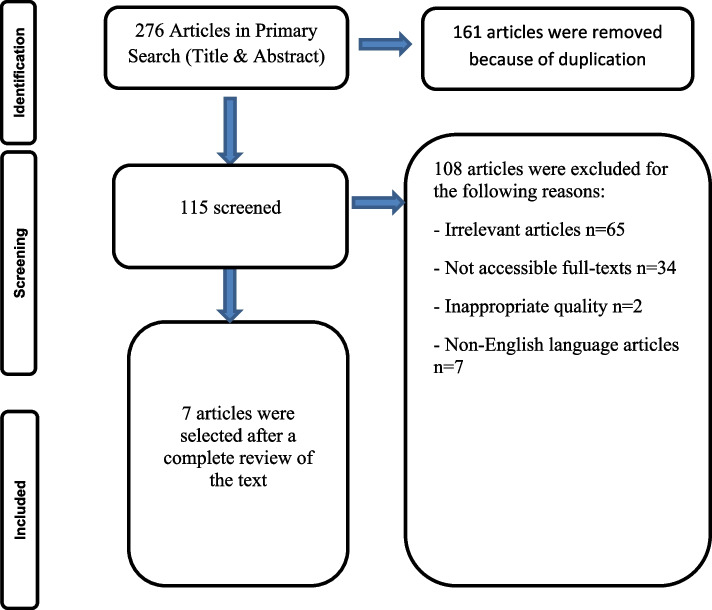
The flow diagram of the review

In all, 276 citations were identified (Embase=62, Web of Science=48, Scopus=82, PubMed=84). After reviewing the title, duplicate cases were removed (*n*=161). The remaining citations were assessed through the abstract and full text of the papers considering inclusion and exclusion criteria and an additional 108 irrelevant articles were excluded. Eventually, 7 full-text articles satisfied the inclusion criteria. The flow diagram of the review is shown in Fig 1.

The first article on the relationship between HPV and vitamin D was published in 2015 [23]. In all studies, vitamin D was assessed by measuring serum 25-hydroxyvitamin D levels [20, 21, 23, 24, 28–30]. Furthermore, 4 emerging biomarkers, (1,25(OH)2D; 24,25(OH)2D; free vitamin D; and vitamin D binding protein) were measured in two studies [29, 30]. One study assessed the short-term persistence of high-risk HPV [29], while other studies investigated transient or sporadic detection of high-risk HPV [20, 21, 23, 24, 28, 30]. The HPV DNA testing was followed by self-collection cervicovaginal swab specimens [20, 24, 29, 30]; cervical sample and colposcopic investigations [21]; Pap smear and HPV test [23, 28]. All studies investigated high-risk HPV patients. However, two studies considered both high-risk and low-risk patients [20, 24]. One study was conducted on a sample of women with systemic lupus erythematosus [23].

According to three studies, there was a significant relationship between vitamin D deficiency and cervicovaginal HPV [20, 21, 28], while three studies did not show any relationship [23, 24, 30]. One study showed a significant positive association between serum vitamin D measured by multiple biomarkers and short-term high-risk HPV persistence [29]. However, they emphasized that the relationship between vitamin D status, as measured by 5 biomarkers, and short-term persistence of high-risk HPV led to mixed results [29]. Also, a report showed that higher levels of a new biomarker 24,25(OH)2D3 were positively associated with a higher likelihood of high-risk HPV detection (aOR ¼ 1.22; 95% CI, 0.97–1.52) [30], whereas the study of Ozgu et al. (2016) showed that deficiency of Vitamin D metabolites can be a possible reason for HPVDNA persistence and related cervical intraepithelial neoplasia (*P*=0.009) [21]. Table 3 demonstrates data extracted from the included Studies.

**Table 3:** Data extracted from the included studies

**Author (Year)/Ref.**	**Country**	**Design**	**Sample size/Participants**	**Findings**
Garcia-Carrasco et al. (2015) [23]	Mexico	Cross-sectional	67 / patients with systemic lupus erythematous	No significant relationship was found between vitamin D deficiency and cervical HPV (*P*=0.7).
Shim et al. (2016) [20]	USA	Cross-sectional	2351 / 20-59 years	Cervicovaginal HPV prevalence was associated with less-than-optimal levels of serum vitamin D (aOR, 1.14; 95% CI, 1.02– 1.27).
Ozgu et al. (2016) [21]	Turkey	Case-control	85 / 20-65 years/ 23 cases: positive HPVDNA testing and abnormal PAP smear result / 62 controls: negative HPV DNA testing and cervical biopsy results	Deficiency of Vitamin D and its metabolites can be a possible reason for HPVDNA persistence and related cervical intraepithelial neoplasia (*P*=0.009).
Troja et al. (2020) [30]	USA	Cross-sectional	404 / 30–50 years	Serum vitamin D levels were not associated with hrHPV prevalence. However higher levels of a subtype [24,25(OH)2D3] was positively associated with the higher likelihood of hrHPV detection (aOR ¼ 1.22; 95% CI, 0.97–1.52). No significant associations were observed for other biomarkers.
El-Zein et al. (2021) [24]	Canada	Cohort	490 / 18-24-years	No evidence of an association between low vitamin D levels and increased HPV prevalence, acquisition, or clearance.
Troja et al. (2021) [29]	USA	Longitudinal cohort	72/ 30–50 years	Significant positive association between higher systemic vitamin D stores and short-term hrHPV persistence.
Chu et al. (2021) [28]	Taiwan	Data were derived from the ongoing prospective cohort of health examinations	7699/ women over 20 years	Vitamin D deficiency was associated with the hrHPV infection of the cervix (*P* < 0.05).

*aOR* Adjusted odds ratiom *CI* Confidence interval, *hrHPV* High-risk HPV

## Discussion

The relationship between HPV infection and serum vitamin D levels has garnered increasing attention due to its potential significance in the context of public health and cervical cancer prevention. In this systematic review, we aimed to consolidate existing evidence to discern any noteworthy associations. However, after careful evaluation of the available studies, the findings showed no clear-cut evidence to suggest vitamin D is associated with HPV, although a few studies claimed this association [20, 21, 28]. One of the most striking observations from our systematic review is the inconsistency in the reported findings across various studies. While some studies suggested a potential inverse association between serum vitamin D levels and HPV infection [20, 21, 28], others failed to establish a significant link [23, 24, 29, 30]. This heterogeneity in results highlights the complex and multifaceted nature of HPV infection and vitamin D metabolism. However, one wonders how to interpret and find an explanation for such observations.

The majority of HPV infections are spontaneously cleared by natural immune responses without leading to cancers, and only a small portion of HPV infections are reported to be persistent, which results in precancerous intraepithelial neoplasia and cancer [3]. Vitamin D is known for its immunomodulatory properties, and a deficiency may theoretically compromise the immune response against viral infections such as HPV [33]. The potential role of vitamin D in protecting and strengthening the immune system is considered in several studies [13, 14, 34]. The majority of immune system cells such as macrophages, lymphocytes, and neutrophils, have vitamin D receptors in their nuclei [35]. Vitamin D makes the physical barriers such as the skin, respiratory tract, and genitourinary tract more resistant to bacteria and viruses by upregulating the proteins that promote tight junctions, gap junctions, and adherens junctions [36]. Thus, sufficient vitamin D levels by strengthening the mucous barriers impair HPV penetration into the basal layer; conversely, insufficient vitamin D levels, by increasing vulnerability against HPV penetration and decreasing the host’s ability to clear the virus, lead to higher incidence of HPV infection [24, 37]. In keeping and maintaining an intact epidermal barrier in the skin, vaginal mucosa, and genitourinary system vitamin D plays a protective and efficient role [38]. Additionally, vitamin D has demonstrated anti-inflammatory and anti-proliferative properties, which could potentially influence the progression and persistence of HPV infection [10]. Numerous studies have reported the positive role of vitamin D in preventing carcinogenic processes and decreasing the risk of cancer by viral infections, especially DNA viruses such as HPV [33, 39–41]. An optimal level of vitamin D might exert beneficial effects in the early phases of cervical cancer by preventing its onset and progression [42]. Nevertheless, according to some studies, vitamin D supplementation does not significantly increase the rate of HPV regression [43, 44].

## Excluded evidence

Two studies were not evaluated in the present study according to the inclusion criteria [31, 32]. One study compared the vitamin D levels in 30 HPV-negative and 68 HPV-positive patients and found no statistically significant difference in vitamin D mean levels between the two groups [31]. Similarly, another study compared the vitamin D levels in 94 HPV-negative and 39 HPV-positive patients and no significant difference was observed [32]. The source titles that published these studies Apparently, both studies failed to provide sound evidence since not enough sample sizes were studied, and it seems that the power for such a comparison in both studies was very poor.

## Strengths and limitations

This review included all studies that reported the relationship between vitamin D serum levels in various study populations (reproductive ages, suffering from low-risk and high-risk types of HPV, with abnormal Pap smear specimens, and suffering from systemic lupus erythematosus) and not merely one particular group. However, given the diverse study designs and study populations represented in this review, it is difficult to infer causality. Furthermore, variations in measurement techniques for serum vitamin D levels or its metabolite substances may have contributed to the inconsistency in findings. Moreover, the role of population characteristics, including age, gender, geographical location, and baseline health status of the study participants, might influence vitamin D metabolism and immune response to HPV that were not possible to examine in this review. Finally, considering the design of studies under review, one should note that the evidence derived from cohort studies and a case-control study should receive more weight than the evidence derived from cross-sectional studies. Thus, although not sharply, one might argue that the findings were in favor of a positive association rather than no relationship.

## Conclusions

The systematic review presented here has thoroughly examined the existing literature on the relationship between cervicovaginal HPV infection and serum vitamin D levels. Despite the initial interest and the potential biological plausibility of such a relationship, the findings showed no firm evidence for any association between HPV infection and serum vitamin D levels. This inconclusiveness underscores the need for further well-designed studies to explore this topic comprehensively.

## Data Availability

All data generated during this study are included in this published article.

## Abbreviations

HPV: Human papillomavirus
STD: Sexually transmitted disease

## References

[1] Bray F, Ferlay J, Soerjomataram I, Siegel RL, Torre LA, Jemal A (2018). Global cancer statistics 2018: GLOBOCAN estimates of incidence and mortality worldwide for 36 cancers in 185 countries. CA Cancer J Clin.

[2] Rosales R, Rosales C (2014). Immune therapy for human papillomaviruses-related cancers. World J Clin Oncol.

[3] Okunade KS (2020). Human papillomavirus and cervical cancer. J Obstet Gynaecol.

[4] Seong J, Ryou S, Lee J, Yoo M, Hur S, Choi B-S (2021). Enhanced disease progression due to persistent HPV-16/58 infections in Korean women: a systematic review and the Korea HPV cohort study. Virol J.

[5] Choi S, Ismail A, Pappas-Gogos G, Boussios S (2023). HPV and cervical cancer: A review of epidemiology and screening uptake in the UK. Pathogens.

[6] Sung H, Ferlay J, Siegel RL, Laversanne M, Soerjomataram I, Jemal A (2021). Global cancer statistics 2020: GLOBOCAN estimates of incidence and mortality worldwide for 36 cancers in 185 countries. CA Cancer J Clin.

[7] Stelzle D, Tanaka LF, Lee KK, et al. Estimates of the global burden of cervical cancer associated with HIV. Lancet Glob Health 2020; published online Nov 16. S2214-109X(20)30459-9 10.1016/S2214-109X(20)30459-9 PMC7815633 33212031

[8] Momenimovahed Z, Mazidimoradi A, Maroofi P, Allahqoli L, Salehiniya H, Alkatout I (2023). Global, regional and national burden, incidence, and mortality of cervical cancer. Cancer Rep.

[9] Kombe Kombe AJ, Li B, Zahid A, Mengist HM, Bounda GA, Zhou Y, Jin T (2021). Epidemiology and burden of human papillomavirus and related diseases, molecular pathogenesis, and vaccine evaluation. Front Public Health.

[10] Ono A, Koshiyama M, Nakagawa M, Watanabe Y, Ikuta E, Seki K, Oowaki M (2020). The preventive effect of dietary antioxidants on cervical cancer development. Medicina (Kaunas).

[11] Lopes RDVC, Teixeira JA, Marchioni D, Villa LL, Giuliano AR, Luiza Baggio M, Fisberg RM (2017). Dietary intake of selected nutrients and persistence of HPV infection in men. Int J Cancer.

[12] Albergamo A, Apprato G, Silvagno F (2022). The role of vitamin D in supporting health in the COVID-19 Era. Int J Mol Sci.

[13] Colotta F, Jansson B, Bonelli F (2017). Modulation of inflammatory and immune responses by vitamin D. J Autoimmun.

[14] Di Rosa M, Malaguarnera M, Nicoletti F, Malaguarnera L (2011). Vitamin D3: a helpful immuno-modulator. Immunology.

[15] Weir EK, Thenappan T, Bhargava M, Chen Y (2020). Does vitamin D deficiency increase the severity of COVID-19?. Clin Med (Lond).

[16] Wang H, Chen W, Li D, Yin X, Zhang X, Olsen N, Zheng SG. Vitamin D and chronic diseases. Aging Dis. 2017; 8(3):346-353. 10.14336/AD.2016.1021. PMID: 28580189. 10.14336/AD.2016.1021 PMC5440113 28580189

[17] Teymoori-Rad M, Shokri F, Salimi V, Marashi SM (2019). The interplay between vitamin D and viral infections. Rev Med Virol.

[18] Wei R, Christakos S (2015). Mechanisms underlying the regulation of innate and adaptive immunity by vitamin D. Nutrients.

[19] Yamshchikov AV, Desai NS, Blumberg HM, Ziegler TR, Tangpricha V (2009). Vitamin D for treatment and prevention of infectious diseases: a systematic review of randomized controlled trials. Endocr Pract.

[20] Shim J, Pérez A, Symanski E, Nyitray AG (2016). Association between serum 25-hydroxyvitamin D level and human papillomavirus cervicovaginal infection in women in the United States. J Infect Dis.

[21] Özgü E, Yılmaz N, Başer E, Güngör T, Erkaya S, Yakut Hİ (2016). Could 25-OH vitamin D deficiency be a reason for HPV infection persistence in cervical premalignant lesions?. J Exp Ther Oncol.

[22] El Mongy NN, Hilal RF, Badr AM, Alraawi SA (2018). Serum vitamin D level in patients with viral warts. J Egypt Women's Dermatologic Soc.

[23] García-Carrasco M, Mendoza-Pinto C, Munguía-Realpozo P (2015). Lack of association between serum 25-hydroxyvitamin D levels and cervical human papillomavirus infection in systemic lupus erythematosus. Lupus.

[24] El-Zein M, Khosrow-Khavar F, Burchell AN, Tellier PP, Eintracht S, McNamara E, Coutlée F, Franco EL; HITCH study group. Association of serum 25-hydroxyvitamin D with prevalence, incidence, and clearance of vaginal HPV infection in young women. J Infect Dis. 2021; 224(3):492-502. 10.1093/infdis/jiaa758. 10.1093/infdis/jiaa758 PMC8328206 33306088

[25] Page M J, McKenzie J E, Bossuyt P M, Boutron I, Hoffmann T C, Mulrow C D et al. The PRISMA 2020 statement: an updated guideline for reporting systematic reviews BMJ 2021; 372 :n71. 10.1136/bmj.n71. 10.1136/bmj.n71 PMC8005924 33782057

[26] Lo CK, Mertz D, Loeb M (2014). Newcastle-Ottawa Scale: comparing reviewers' to authors' assessments. BMC Med Res Methodol.

[27] Wells GA, Shea B, O’Connell D, Peterson J, Welch V, Losos M, et al. The Newcastle-Ottawa Scale (NOS) for assessing the quality if nonrandomized studies in meta-analyses. Available from: URL: http://www.ohri.ca/programs/clinical_epidemiology/oxford.htm.

[28] Chu TW, Jhao JY, Lin TJ, Lin TW, Wang CL, Chang HS, Liu LC, Chang CC (2021). Vitamin D in gynecological diseases. J Chin Med Assoc.

[29] Troja C, Hoofnagle AN, Szpiro A, Stern JE, Lin J, Winer RL (2021). Understanding the role of emerging Vitamin D biomarkers on short-term persistence of high-risk human papillomavirus infection among mid-adult women. J Infect Dis.

[30] Troja C, Hoofnagle AN, Szpiro A, Stern JE, Lin J, Winer RL (2020). Serum concentrations of emerging vitamin D biomarkers and detection of prevalent high-risk HPV infection in mid-adult women. Cancer Epidemiol Biomarkers Prev.

[31] Mertoğlu C, Naykı Ü, Naykı C, Günay M (2017). The relationship between vitamin D And human papilloma virus infection. J Clin Anal Med.

[32] Çakir AT, Özten MA. Serum vitamin D levels in high-risk HPV infected patients, is there any relation? J Clin Med Kaz. 2022; 19(3):35-39. 10.23950/jcmk/12113.

[33] Siddiqui M, Manansala JS, Abdulrahman HA, Nasrallah GK, Smatti MK, Younes N, Althani AA, Yassine HM (2020). Immune modulatory effects of vitamin D on viral infections. Nutrients.

[34] Beard JA, Bearden A, Striker R (2011). Vitamin D and the anti-viral state. J Investig Med.

[35] Gallo D, Baci D, Kustrimovic N, Lanzo N, Patera B, Tanda ML, Piantanida E, Mortara L (2023). How does vitamin D affect immune cells crosstalk in autoimmune diseases?. Int J Mol Sci.

[36] Sun J, Zhang YG (2022). Vitamin D receptor influences intestinal barriers in health and disease. Cells.

[37] Hewavisenti RV, Arena J, Ahlenstiel CL, Sasson SC (2023). Human papillomavirus in the setting of immunodeficiency: Pathogenesis and the emergence of next-generation therapies to reduce the high associated cancer risk. Front Immunol.

[38] Piotrowska A, Wierzbicka J, Żmijewski MA (2016). Vitamin D in the skin physiology and pathology. Acta Biochim Pol.

[39] Fleet JC, DeSmet M, Johnson R, Li Y (2011). Vitamin D and Cancer: A review of molecular mechanisms. Biochem J.

[40] Gunville CF, Mourani PM, Ginde AA (2013). The role of vitamin D in prevention and treatment of infection. Inflamm Allergy Drug Targets.

[41] Fathi N, Ahmadian E, Shahi S, Roshangar L, Khan H, Kouhsoltani M, Maleki Dizaj S, Sharifi S (2019). Role of vitamin D and vitamin D receptor (VDR) in oral cancer. Biomed Pharmacother.

[42] Avila E, Noriega-Mejía BJ, González-Macías J, Cortes-Hernández U, García-Quiroz J, García-Becerra R, Díaz L (2023). The preventive role of the vitamin D endocrine system in cervical cancer. Int J Mol Sci.

[43] Zhu G, Li Z, Tang L, Shen M, Zhou Z, Wei Y, Zhao Y, Bai S, Song L (2022). Associations of dietary intakes with gynecological cancers: Findings from a cross-sectional study. Nutrients.

[44] Koc S, Kurt S, Ilgen O, Timur H, Uslu T (2021). The effect of vitamin D on the regression of human papilloma virus infection and metabolic parameters: a retrospective study. Eur J Gynaecol Oncol.

